# Agreement of diameter- and volume-based pulmonary nodule management in CT lung cancer screening

**DOI:** 10.1186/1470-7330-14-S1-S4

**Published:** 2014-10-09

**Authors:** MA Heuvelmans, R Vliegenthart, N Horeweg, GJ de Jonge, PMA van Ooijen, PA de Jong, ETh Scholten, GH de Bock, WPTM Mali, HJ de Koning, M Oudkerk

**Affiliations:** 1 Center for Medical Imaging – North East Netherlands, Department of Radiology, University of Groningen, University Medical Center Groningen, Groningen, Netherlands; 2 Department of Public Health, Erasmus MC, Rotterdam, Netherlands; 3 Department of Radiology, University Medical Center Utrecht, Utrecht, Netherlands; 4 Department of Epidemiology, University of Groningen, University Medical Center Groningen, Groningen, Netherlands

## Aim

To determine the agreement of manual and semi-automatic (SA) diameter and volume measurements of nodules found in low-dose computed tomography lung cancer screening.

## Methods

Baseline data of 2,240 solid intermediate-sized nodules (volume 50-500mm^3^) in 1,498 Dutch-Belgian NELSON trial participants were used. Extrapolated volume based on semi-automatic (SA) maximum diameter and mean of maximum transversal and perpendicular diameter were compared to SA volume measurements by Bland-Altman plots. Analyses were repeated by margin (smooth, lobulated, spiculated, and irregular) and shape (spherical or non-spherical). In 100 randomly selected nodules, diameters were measured manually by two independent radiologists, and compared to the SA diameters.

## Results

Median participant age was 59-years (interquartile range:8), 14.2% were women. Compared to SA volume, volume extrapolated from SA mean or maximum diameter led to mean overestimation of 47.2% (95%-confidence interval (CI): 44.7-49.7%) and 85.1% (95%-CI:81.2-89.0%), respectively. For irregular and non-spherical nodules, mean overestimation was higher; 161.7% (95%-CI:131.7%-191.8%) and 168.9% (95%-CI:155.2%-182.5%), respectively. Manual diameter measurement overestimated SA maximum diameter by ≥10% in 44% (44/100) and underestimated by ≥10% in 18% (18/100) of the nodules. Using a 10-mm criterion for referral, SA maximum diameter measurements of indeterminate nodules would have led to direct referral in 7.9% (177/2240). Manual measurements would have led to 31% (31/100) referrals.

## Conclusion

The agreement between manual and SA diameter, as well as between volume extrapolated from SA diameter and SA volume is poor. Applying manual and SA diameter measurement in CT lung cancer screening leads to a substantial shift in nodule stratification compared to SA volume measurements.

